# Proposal for individualized dosing of eculizumab in atypical haemolytic uraemic syndrome: patient friendly and cost-effective

**DOI:** 10.1093/ndt/gfac056

**Published:** 2022-03-03

**Authors:** Mendy ter Avest, Romy N Bouwmeester, Caroline Duineveld, Kioa L Wijnsma, Elena B Volokhina, Lambertus P W J van den Heuvel, David M Burger, Jack F M Wetzels, Nicole C A J van de Kar, Rob ter Heine, CUREiHUS study group, E van Kempen, W Altena, E Adang, D J A R Moes, A D van Zuijlen, S P Berger, F J Bemelman, J W van der Heijden, J van de Wetering, A P J de Vries, P van Paasen, J F M Wetzels, J A E van Wijk, A H M Bouts, E M Dorresteijn, V Gracchi, F A P T Horuz Engels, M G Keijzer-Veen, R W G van Rooij, N C A J van de Kar

**Affiliations:** Department of Pharmacy, Radboud Institute for Health Sciences, Radboud University Medical Centre, Nijmegen, The Netherlands; Department of Paediatric Nephrology, Radboud Institute for Molecular Life Sciences, Amalia Children's Hospital, Radboud University Medical Centre, Nijmegen, The Netherlands; Department of Nephrology, Radboud University Medical Centre, Nijmegen, The Netherlands; Department of Paediatric Nephrology, Radboud Institute for Molecular Life Sciences, Amalia Children's Hospital, Radboud University Medical Centre, Nijmegen, The Netherlands; Department of Paediatric Nephrology, Radboud Institute for Molecular Life Sciences, Amalia Children's Hospital, Radboud University Medical Centre, Nijmegen, The Netherlands; Department of Laboratory Medicine, Radboud University Medical Centre, Nijmegen, The Netherlands; Department of Paediatric Nephrology, Radboud Institute for Molecular Life Sciences, Amalia Children's Hospital, Radboud University Medical Centre, Nijmegen, The Netherlands; Department of Laboratory Medicine, Radboud University Medical Centre, Nijmegen, The Netherlands; Department of Pharmacy, Radboud Institute for Health Sciences, Radboud University Medical Centre, Nijmegen, The Netherlands; Department of Nephrology, Radboud University Medical Centre, Nijmegen, The Netherlands; Department of Paediatric Nephrology, Radboud Institute for Molecular Life Sciences, Amalia Children's Hospital, Radboud University Medical Centre, Nijmegen, The Netherlands; Department of Pharmacy, Radboud Institute for Health Sciences, Radboud University Medical Centre, Nijmegen, The Netherlands

**Keywords:** aHUS, complement, eculizumab, pharmacodynamics, pharmacokinetics

## Abstract

**Background:**

Eculizumab is a lifesaving yet expensive drug for atypical haemolytic uraemic syndrome (aHUS). Current guidelines advise a fixed-dosing schedule, which can be suboptimal and inflexible in the individual patient.

**Methods:**

We evaluated the pharmacokinetics (PK) and pharmacodynamics (PD) [classical pathway (CP) activity levels] of eculizumab in 48 patients, consisting of 849 time-concentration data and 569 CP activity levels. PK–PD modelling was performed with non-linear mixed-effects modelling. The final model was used to develop improved dosing strategies.

**Results:**

A PK model with parallel linear and non-linear elimination rates best described the data with the parameter estimates clearance 0.163 L/day, volume of distribution 6.42 L, maximal rate 29.6 mg/day and concentration for 50% of maximum rate 37.9 mg/L. The PK–PD relation between eculizumab concentration and CP activity was described using an inhibitory *E*_max_ model with the parameter estimates baseline 101%, maximal inhibitory effect 95.9%, concentration for 50% inhibition 22.0 mg/L and  Hill coefficient 5.42. A weight-based loading dose, followed by PK-guided dosing was found to improve treatment. On day 7, we predict 99.95% of the patients to reach the efficacy target (CP activity <10%), compared with 94.75% with standard dosing. Comparable efficacy was predicted during the maintenance phase, while the dosing interval could be prolonged in ∼33% of the population by means of individualized dosing. With a fixed-dose 4-week dosing interval to allow for holidays, treatment costs will increase by 7.1% and we predict 91% of the patients will reach the efficacy target.

**Conclusions:**

A patient-friendly individualized dosing strategy of eculizumab has the potential to improve treatment response at reduced costs.

KEY LEARNING POINTS **What is already known about this subject?**

- Eculizumab is a very expensive yet lifesaving drug for atypical haemolytic uraemic syndrome.
- Current guidelines advise a fixed dosing schedule, which can be suboptimal and inflexible in the individual patient.

**What this study adds?**

- This article describes the development of a pharmacokinetic–pharmacodynamic model of eculizumab that was subsequently used to explore alternative dosing regimens to improve treatment efficacy, patient friendliness and cost-effectiveness.

**What impact this may have on practice or policy?**

- Individualized dosing of eculizumab results in a more patient-friendly dosing regimen at reduced costs.
- We show the feasibility of increasing dosing intervals to a fixed-dose 4-week interval regimen to allow for holidays.

## INTRODUCTION

Atypical haemolytic uraemic syndrome (aHUS) is a rare variant of thrombotic microangiopathy and is characterized by mechanical haemolytic anaemia, thrombocytopenia and ischaemia in end organs such as acute kidney injury. aHUS is caused by overactivation of the complement alternative pathway due to mutations in complement genes or acquired autoantibodies directed against complement factor H. This complement dysregulation leads to complement deposition on endothelial cells, causing endothelial cell activation and injury [1, 2].

With the introduction of eculizumab as a treatment for aHUS, mortality and morbidity were drastically reduced [3, 4]. Eculizumab is a humanized monoclonal antibody that binds complement factor C5, inhibiting the C5 cleavage of C5a and C5b and subsequently the formation of the terminal complement complex C5b–9 [5]. Recently the long-acting C5 inhibitor ravulizumab-cwvz has been approved by the European Medicines Agency (EMA) and the Food and Drug Administration to treat patients with aHUS as well [6, 7]. Currently eculizumab is the only drug adopted in the treatment guidelines of aHUS in the Netherlands. Like other orphan drugs, therapy with eculizumab is expensive with costs up to US$550 000/patient/year following dosing according to the drug label [8].

Following the drug label, eculizumab therapy consists of two phases, an initial phase and a maintenance phase. For adult patients and children weighing ˃40 kg, eculizumab is dosed in a flat fixed dose for every patient. Children weighing <40 kg are treated with a weight-based dosing regimen (Table 1) [9].

**Table 1.** Approved eculizumab dosing regimen

	Induction phase				Maintenance phase				
Group	Week, dose				Week, dose				Interval
Adults and children ≥40 kg	1 900 mg	2 900 mg	3 900 mg	4 900 mg	5 1200 mg	6 -	7 1200 mg	8 -	Every 14 days
Children 30–40 kg	1 600 mg		2 600 mg		3 900 mg	4 -	5 900 mg	6 -	
Children 20–30 kg	1 600 mg		2 600 mg		3 600 mg	4 -	5 600 mg	6 -	
Children 10– 20 kg	1 600 mg				2 300 mg	3 -	4 300 mg	5 -	
Children 5–10 kg	1 300 mg				2 300 mg	3 -	4 -	5 300 mg	Every 21 days

As anti-C5 therapy like eculizumab is the only treatment for patients with aHUS, optimizing treatment is indispensable. The drug label states that a trough concentration of 50–100 mg/L is sufficient for complete inhibition of the terminal complement complex [9]. However, two important issues with eculizumab treatment need to be addressed.

First, with the approved loading dose treatment scheme, exposure is often subtherapeutic after the first dose [10], while in early treatment, adequate therapy is of utmost importance to prevent thrombotic microangiopathy and chronic sequelae [11, 12]. Additionally, a weekly treatment scheme in the initial phase is less patient-friendly, especially for outpatients.

Furthermore, supratherapeutic eculizumab concentrations are often observed in the maintenance phase, explained by the large interindividual variability in pharmacokinetics (PK) [4, 13–19]. Therefore, dose interval prolongation might be an option for individual patients. Additionally, a 4-week interval might be preferable for all patients during holidays.

Like other monoclonal antibodies, eculizumab has a wide therapeutic range and no concentration toxicity relationship has been observed [20, 21]. However, from a societal perspective, the high costs of eculizumab treatment dictate that one should aim to avoid unnecessary overexposure to eculizumab. An individualized treatment approach may help to improve patient-friendliness, maximize treatment response and reduce treatment costs. The starting point for truly tailored eculizumab dosing is the development of a population PK–pharmacodynamic (PD) model. In this study we developed such a model and explored alternative dosing regimens to improve early treatment response and patient-friendliness at, preferably, lower costs.

## MATERIALS AND METHODS

### Study design and population

This study was an add-on study of the National observational study to monitor the new guideline concerning the treatment of patients with atypical haemolytic uraemic syndrome (CUREiHUS; NTR5988/NL5833) [22], approved by the local human research and ethics committee and was conducted in accordance with the principles of the Declaration of Helsinki.

Paediatric and adult aHUS patients in the Netherlands who were treated following the new Dutch guideline (2016, 2019) concerning the treatment of aHUS and who provided informed consent were included in the CUREiHUS study. Patients were treated with eculizumab with the standard dose for 3 months. When in clinical remission, therapy was discontinued in patients ˃6 years of age and the dose was optionally tapered in children ˂6 years of age. Therapy was continued when clinical remission was not obtained or restarted and optionally subsequently continued after relapse of aHUS. Eculizumab trough concentrations and complement activation markers were measured as part of the CUREiHUS study or as part of routine patient care. After separate informed consent for the add-on study, additional blood samples between eculizumab administrations were drawn at 2–4, 24, 72 and 120 h after administration. Patients were included in our PK–PD study if at least one eculizumab concentration was measured during eculizumab treatment.

### Bioanalysis of eculizumab concentrations and classical pathway activity

Free eculizumab concentrations were measured by using a validated enzyme-linked immuno sorbent assay (ELISA) [13] or by a validated ELISA at Sanquin, Amsterdam, the Netherlands. Classical complement pathway (CP) activity was measured by using a commercial Wieslab complement system screen (Euro Diagnostica, Malmö, Sweden) [17] or by an in-house developed and validated ELISA [13]. CP activity is expressed as a percentage of a range of control sera [23]. A CP percentage <10% was considered to be equal to complete complement blockade. Method comparison for both the eculizumab assays and the CP activity assays was done pair-wise for available data. Passing–Bablok regression analysis was used to compare the methods. Bland–Altman plots were used to measure agreement between the methods.

### Development of a PK model

Population PK modelling was performed with NONMEM version 7.4.3 (ICON Development Solutions, Dublin, Ireland). Single and multiple-compartment models were tested with both first-order elimination and combined first-order and Michaelis–Menten elimination. The detailed description of the development of the PK model can be found in the supplemental data. To evaluate the predictive performance of the population PK model, a prediction-corrected visual predictive check (pcVPC) was made, based on 1000 Monte Carlo simulations (Figure 2). The principle of a VPC is to assess graphically whether simulations from the model are able to reproduce the central trend and variability in observed data when plotted versus an independent variable (in this case time after dose) [24].

### Development of a PK–PD model

After establishing the model that best described the PK of eculizumab, a sequential PK–PD model was developed to describe the relationship between the free eculizumab concentration and the degree of complement blockade. An inhibitory *E*_max_ model was used to construct this relationship:

$$\begin{equation*}E = Base * \left( {1 - \frac{{{I_{\rm Max}} * {C^\gamma }}}{{IC_{50}^\gamma + {C^\gamma }}}} \right),\end{equation*}$$

where *E* is the complement inhibitory effect of eculizumab, *Base* is the initial classical pathway activity in the absence of eculizumab, *I*_max_ is the maximal inhibitory effect of eculizumab, *C* is the free eculizumab concentration, *IC*_50_ is the free eculizumab concentration for 50% classical pathway activity inhibition and γ is the Hill coefficient. A detailed description of the development of the PK–PD model is provided in the supplemental data.

### Exploration of alternative dosing strategies of eculizumab: a simulation study

The final PK–PD model was used to investigate alternative dosing strategies through Monte Carlo simulations. To obtain a representative population, a dataset with 2000 individuals ages 1–80 years was derived from the National Health and Nutrition Examination Survey (NHANES) database [25]. This cohort of the NHANES database consisted of 48% females, a median weight of 61.6 kg (range 8.3–155.6), median age of 24.8 years (range 1–79) and median height of 159 cm (range 70–202), which was comparable with our aHUS population with respect to these characteristics.

The alternative regimens were chosen at the discretion of the researcher. The optimal strategy was defined as the regimen with the highest percentage of individuals with effective complement blockade (CP activity <10%), without increasing the cumulative dose if possible. For each scenario, we predicted eculizumab concentrations and classical pathway activity. To predict the dosing costs, we assumed costs of US$6523 per eculizumab vial of 300 mg [26].

#### Loading dose

We aimed to develop a dosing regimen with a single loading dose, followed by the maintenance dose on day 15 of treatment.

#### Individualized dosing of eculizumab in the maintenance phase

Subsequently, a new maintenance phase dosing strategy was investigated. To determine the optimal maintenance dosing regimen, we simulated the effect of extending the dosing interval or increasing the dose based on trough level measurements before the second and third dose.

#### Fixed-dose 4-week interval of eculizumab

In clinical practice, extended dosing intervals can be useful to allow holidays during treatment. As therapeutic drug monitoring (TDM) of eculizumab cannot be performed in every clinic, we investigated if the dosing interval of eculizumab could be extended to 4 weeks by increasing the dose.

## RESULTS

### Demographics and data

In total, 48 aHUS patients treated with eculizumab with at least one available PK sample were included in this study, with a total of 849 paired observations of time and free eculizumab concentrations at a median of 12 occasions (range 1–93) and 569 CP activity levels. Patient characteristics at baseline are summarized in Table 2.

**Table 2.** Baseline characteristics

Characteristics	Adults (*n* = 38)	Children (*n* = 10)	All (*n* = 48)
Age (years), median (IQR) [range]	43 (31–48) [21–78]	8 (2–11) [1–12]	39 (23–47) [1–78]
Sex (female), *n* (%)	27 (71)	5 (50)	32 (66.6)
Weight (kg), median (IQR) [range]	76.6 (65.0–87) [54.2–106.8]	27.5 (14.5–45.3) [10.7–52.6]	71.9 (54.9–84.4) [10.7–106.8]
Length (cm), median (IQR) [range]	170 (165–178) [154–200]	130 (93–152) [82–166]	168 (159–175) [82–200]
Eculizumab concentration (mg/L) (*n* = 849), median (IQR)			173 (78–302)

### Bioanalysis of eculizumab concentrations and classical pathway activity

Free eculizumab concentrations were measured with both methods in 29 samples. Passing–Bablok regression revealed that *C*_s_ = 12.66 + 1.14*C*_r._ In this equation, *C*_s_ is the eculizumab concentration measured with the Sanquin method and *C*_r_ is the eculizumab concentration measured with the Radboudumc method [slope 95% confidence interval (CI) 0.96–1.32; *y*-intercept 95% CI −56.78–31.45]. Since the slope and intercept CI included 1 and 0, respectively, no structural bias was observed and an additional residual error was estimated for each bioanalytical method, to allow simultaneous analysis of the PK data (Supplementary data, Figure S1).

CP activity levels were measured with both methods in 60 samples. Passing–Bablok regression showed that CP_LMI_ = 6.33 + 1.05*CP_Wieslab_ (slope 95% CI 0.966–1.131; *y*-intercept 95% CI 0.003–12.63). In this equation, CP_LMI_ is the CP activity measured with our ‘in-house’ method and CP_Wieslab_ is the CP activity measured with Wieslab ELISA. The Passing–Bablok regression analysis revealed a significant difference between both methods in the *y*-intercept. Therefore we converted our ‘in-house’ ELISA values to Wieslab ELISA values using the relationship described above (see also Supplementary data, Figure S2).

### Development of a population PK model

**FIGURE 1:**
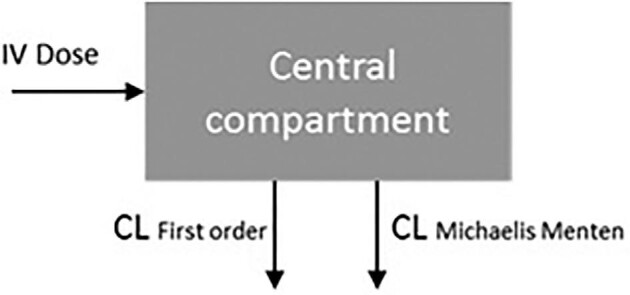
Graphical display of the pharmacokinetic model of eculizumab.

A one-compartment model, integrally describing the PK of eculizumab in adults and in children, with parallel first-order and Michaelis–Menten elimination best described the data. Figure 1 schematically shows the model. Population values for clearance (*CL*), volume of distribution (*V*_d_), maximum rate (*V*_max_) and plasma concentration for 50% of maximum rate (*K*_m_) for a typical person of 70 kg were estimated to be 0.163 L/day [relative standard error % (RSE) 7.5], 6.42 L (5.9), 29.6 mg/day (7.0) and 37.9 mg/L (18.7) (Table 3). The supplementary data describe the results in more detail.

**FIGURE 2:**
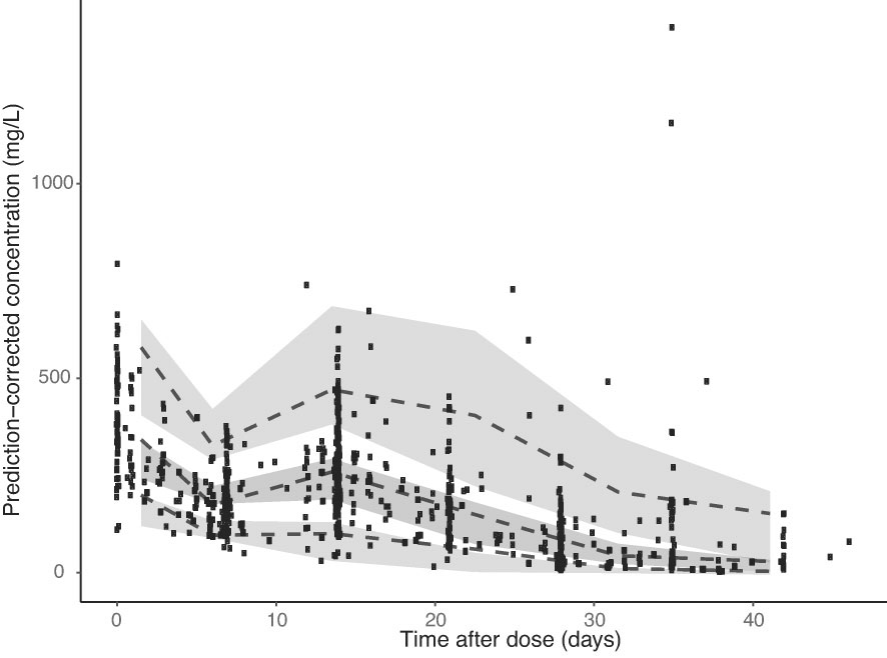
Prediction-corrected visual predictive check of the final PK model of eculizumab. The black dots represent the observed concentrations. The dashed lines represent the 5th, median, 95th percentile of the predictions. The shaded grey areas represent the corresponding 95% CIs. The majority of the predicted concentrations are in line with the observed concentrations, indicating appropriate validity of the model.

**Table 3.** Population estimates for the final PK model

Parameter	Estimate (RSE%)	IIV (CV%) (RSE%)	IOV (CV%) (RSE%)
Clearance (L/day)	0.163 (7.5)	43.4 (11.4)	34.4 (5.2)
Volume of distribution (L)	6.42 (5.9)	37.1 (12.5)	
Maximum rate (*V*_max_) (mg/day)	29.6 (7.0)		
Plasma concentration for 50% of maximum rate (*K*_m_) (mg/L)	37.9 (18.7)		
Additional error (mg/L)	4.33 (46.3)		
Proportional error Radboud Sanquin	0.0247 (3.7) 0.248 (32.4)		

RSE, relative standard error; IIV, interindividual variability; IOV, interoccasion variability.

### Development of a PK–PD model

**FIGURE 3:**
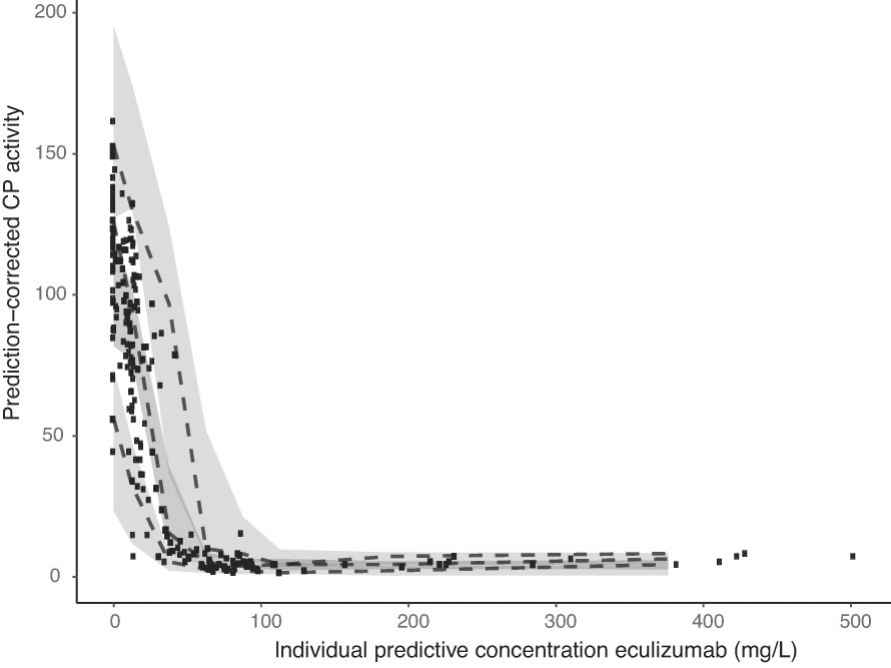
Prediction-corrected visual predictive check for the final model. The black dots represent the observed CP activity at corresponding eculizumab concentrations. The dashed lines represent the 5th, median and 95th percentile of the predictions. The majority of the predicted concentrations are in line with the observed concentrations, indicating appropriate validity of the model.

For the inhibitory *E*_max_ model estimations for *Base, I*_max_, *IC*_50_ and γ were 101% (RSE% 6.2), 95.9% (20), 22.0 mg/L (8.6) and 5.42 (4.6), respectively (Table 4). Figure 3 shows the pcVPC. The supplementary data describe the results in more detail.

**Table 4.** Population estimates for the final sequential PK–PD model

Parameter	Estimate(RSE%)	IIV (CV%) (RSE%)
Baseline CP activity (%)	100.7 (6.2)	23 (19.2)
Maximum inhibition (*I*_max_)	0.96 (0.2)	
Plasma IC_50_ (mg/L)	22.0 (8.6)	38.5 (16.2)
Hill coefficient (γ)	5.42 (4.6)	
Proportional error	0.089 (3.7)	

RSE: relative standard error; IIV, interindividual variability.

### Exploration of alternative dosing strategies of eculizumab

#### Loading dose strategy

**FIGURE 4:**
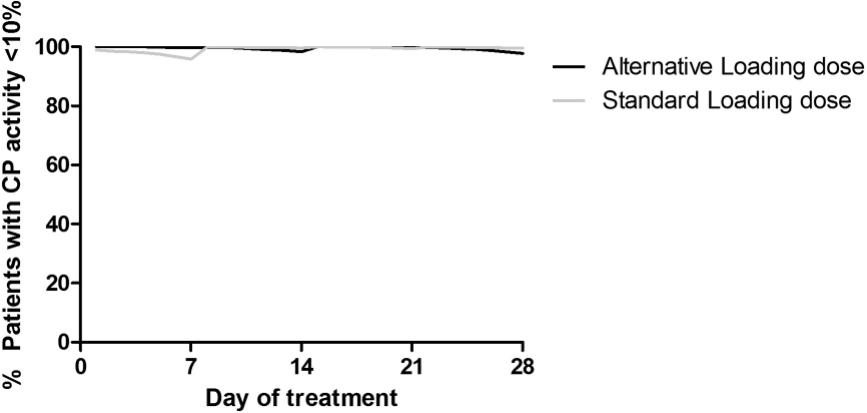
Percentage of patients with a CP activity <10% over time for the standard loading dose (grey line) and alternative loading dose (black line).

Figure 4 shows the percentage of patients with effective complement blockade in the first 28 days of treatment for the standard loading dose and the alternative loading doses (Table 5). On day 7 of therapy, we predicted that with the alternative loading dose, 99.95% of the patients would reach the efficacy target on day 7, compared with 94.75% with standard dosing.

**Table 5.** Alternative loading dose strategy for eculizumab

	Induction phase	Maintenance phase	
Patient weight (kg)	Day 1	Day 15	Beyond
≥120	2400 mg	1200 mg	Standard maintenance dosing (Table 1)
90–120	2100 mg	1200 mg	
60–90	1800 mg	1200 mg	
40–60	1500 mg	1200 mg	
30–40	900 mg	900 mg	
20–30	600 mg	600 mg	
10–20	600 mg	300 mg	
5–10	300 mg	300 mg	

The predicted mean drug costs of the first 28 days of treatment in our cohort of 2000 patients (1–79 years) were US$ 82 128 for the standard loading dose regimen (in case of adults: 4× loading dose, 1× maintenance phase dose) and US$71 678 for the alternative dose regimen (1× loading dose, 2× standard maintenance phase dose), showing a potential of ∼13% reduction in drug costs in the first 28 days of treatment. With this alternative dosing regimen, only three infusions of eculizumab have to be administered in the first 28 days of treatment compared with five infusions in the standard dosing regimen.

#### Individualized dosing of eculizumab in the maintenance phase

**FIGURE 5:**
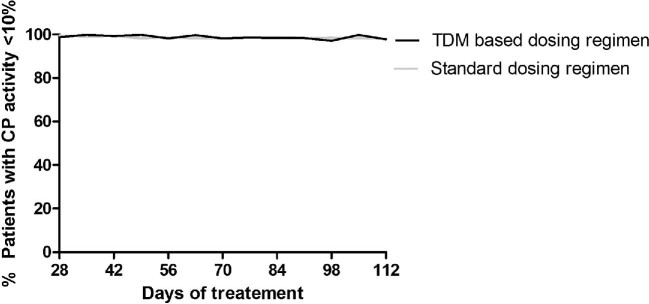
Percentage of patients with a CP activity <10% over time for the standard dosing regimen (grey line) and an individualized, TDM-based dosing regimen (black line).

Figure 5 shows the percentage of patients with effective complement inhibition in the maintenance phase of treatment for the standard and the individualized dosing regimen (Table 6).

**Table 6.** Alternative maintenance dosing strategy for eculizumab

		Dose adjustment	
**C_trough_ (2nd dose)**	**C_trough_ (3rd dose)**	**Interval**	**Dose**
<100	<50	Unchanged	+300 mg
100–200	50–200	Unchanged	Unchanged
≥200	≥200	+1 week	Unchanged

Comparable percentages of target attainment were predicted during the maintenance phase for both standard and individualized dosing regimens (97.5% versus 96.5%). Also, comparable eculizumab trough concentrations were predicted between 50 and 100 mg/L (10.5% versus 12.8%). The dosing interval could be extended in ∼33% of patients [3 weeks (26.8%), 4 weeks (6.8%)] without changing the dose. Overall, the mean yearly maintenance eculizumab drug costs for the standard maintenance dosing regimen are US$ 537 514 compared with US$ 514 696 for the individualized dosing regimen, showing a potential ∼4.2% cost reduction in the maintenance phase, while increasing patient-friendliness.

#### Fixed-dose 4-week dosing interval of eculizumab

**FIGURE 6:**
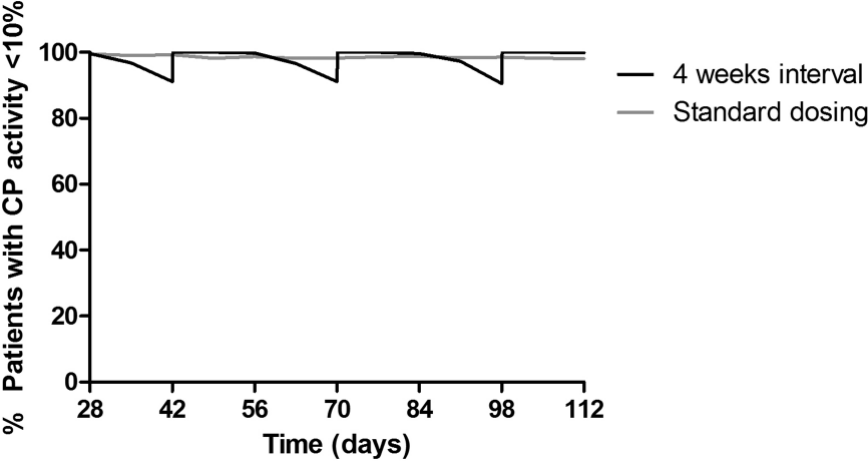
Percentage of patients with a CP activity <10% over time for the standard dosing regimen (grey line) and a standard 4-week interval (black line).

Figure 6 shows the percentage of patients with effective complement blockade in the maintenance phase of treatment for the standard maintenance phase dosing regimen and for a 4-week interval (see Table 7 for the most optimal 4-week interval strategy). For the 4-week interval, 100, ∼97 and ∼91% of all patients are predicted to have a CP activity <10% at 2, 3 and 4 weeks after the last dose, respectively. Overall, the mean yearly maintenance eculizumab drug costs for the standard dosing regimen were predicted to be US$537 514 compared with US$575 785 for the 4-week interval. With the 4-week regimen, yearly drug costs will increase by 7.1%.

**Table 7.** Alternative 4-week interval for eculizumab, e.g. around holidays

	Maintenance
Patient weight (kg)	4-week interval
≥120	3000 mg
90–120	2700 mg
60–90	2400 mg
40–60	2100 mg
30–40	1800 mg
20–30	1500 mg
10–20	1200 mg
5–10	900 mg

## DISCUSSION

To our knowledge, this study is the first to describe the PK and PD of eculizumab in aHUS patients. We demonstrated the potential of a new weight-based loading dose strategy for eculizumab, resulting in a larger proportion of patients who reach the efficacy target in the initial treatment phase, with fewer infusions at reduced drug costs. Furthermore, we showed that individualized dosing may be used to prolong the dosing interval in approximately one-third of all patients, while also decreasing treatment costs. Lastly, we show the potential of using a 4-week dosing interval of eculizumab, in which 91% of all patients reach the efficacy target during the complete dosing interval.

We developed our PK–PD model based on data from 48 aHUS patients, consisting of both children and adults. To our knowledge, only three other PK studies were performed with eculizumab [14, 19, 27].

In our study we found a volume of distribution of 6.42 L,  which is comparable with the data in the approval review documents of eculizumab from the EMA and FDA and other monoclonal antibodies [9, 28], but higher than found in other studies [14, 19]. Due to the large molecular weight of eculizumab (148 kDa) [9], a low volume of distribution (3–8 L) at steady state is expected, reflecting the volume of vascular and interstitial spaces [29]. As the measurement of peak concentrations was part of our PK study, we consider the volume of distribution in our model as reliable.

This study confirms previous findings of a highly variable interindividual clearance of eculizumab (43.3%). Factors that may influence the PK and PD of eculizumab have been reviewed before [30]. In our study, body weight was a covariate for clearance and volume of distribution. Elimination of monoclonal antibodies often results from a combination of linear non-specific elimination and receptor-mediated elimination [29]. We found that the Michaelis–Menten constant for clearance was 37.9 mg/L. This corresponds well with our PK–PD analysis, where we found that the receptor affinity (IC_50_) was comparable at 22 mg/L. Furthermore, we found a relatively steep concentration–response curve, with an estimated Hill coefficient of 5.42. These findings are well-aligned with data from the license holder, who found an IC_50_ of 40.8 mg/L and a Hill coefficient of 4.1 [31]. Gatault *et al.* [14] also developed a PK model with a non-linear elimination term, but Passot *et al.* [19] only used a linear elimination rate. In the CUREiHUS study, dosing intervals of eculizumab were individually extended to intervals up to 6 weeks, so we were able to observe low eculizumab concentrations (8–50 mg/L) in the range where target-mediated clearance becomes apparent. This likely explains why we could identify this non-linear receptor-mediated clearance.

In addition, we also observed a high intra-individual variability of eculizumab clearance (34.4% CV). In particular, the non-linear target-mediated elimination of eculizumab can vary over time, due to variations in the amount of available C5 (e.g. due to infection) [32]. Jodele *et al.* [27] reported faster eculizumab clearance when patients had higher sC5b-9 concentrations. We recently showed a case of increased eculizumab clearance that was probably due to increased proteinuria, a condition that is not uncommon in aHUS patients [33]. Due to the high variability in PK of eculizumab, TDM is recommended to optimize therapy. CP activity can also be used to discover potential subtherapeutic eculizumab concentrations, but as residual complement activity (CP >10%) is rarely described in patients with eculizumab concentrations >100mg/L, it cannot be used to discover supratherapeutic concentrations.

By using a weight-based loading dose on day 1 of therapy, we were able to improve early target attainment of eculizumab during the loading dose. We predicted that 99.95% of the patients reach the efficacy target (CP <10%) on day 7 with our new strategy, compared with 94.75% with standard dosing. In addition, only three infusions of eculizumab are necessary compared with five in the standard dosing regimen and we predict potential costs savings of 12.5% in the first 28 days of treatment.

For the maintenance dosing phase, we predicted that with individualized dosing, the interval could be prolonged in ∼33% of all patients. We choose to prolong the dosing interval instead of lowering the doses, to improve both patient-friendliness and treatment costs in the maintenance phase. Although frequent drug dosing will eventually be burdensome for most patients, we think patient-friendliness can be slightly improved with prolonged dosing intervals, as it gives a patient more flexibility and reduces the risks of infusion complications. As mentioned before, large intra-individual variability in the clearance of eculizumab was observed, but in our current simulation study, we only assessed eculizumab concentrations before the second and third dose. We hypothesize that with frequent TDM during treatment with eculizumab, dosing intervals can be further prolonged in a larger proportion of patients and drug costs can be further reduced, although this should be monitored prospectively. TDM necessitates the development and validation of an analytical method for eculizumab and the interpretation of eculizumab concentrations by an expert in PK-guided dosing. The costs for quantification of monoclonal antibodies in the blood are ∼US$20–50 per sample [34]. Considering the potential savings, we consider these costs negligible.

As TDM of eculizumab is not yet implemented in every clinic, we aimed to develop a 4-week dosing interval of eculizumab without the necessity of measuring drug concentrations. With our 4-week dosing interval, 91% of all patients reach the efficacy target during the complete dosing interval.

As lifelong eculizumab administration does not seem a prerequisite for effective treatment of aHUS [22, 35] and one may consider a one-time higher dose to allow effective treatment during a holiday, the cost increments due to longer dosing intervals are limited. Furthermore, one saves outpatient treatment costs when administrating eculizumab every 4 weeks instead of every 2 weeks.

With the recent introduction of the long-acting C5-inhibitor ravulizumab-cwvz, the development of a 4r-week interval regimen of eculizumab has probably become less relevant. However, we think that eculizumab might still be the drug of choice in several subgroups of aHUS (e.g patients who need short-term treatment).

Different analytical methods to measure eculizumab concentrations and classical pathway activity were used in this study for logistical reasons. Although one may argue that using different bioanalytical assays, e.g. resulting in slightly different results for eculizumab concentrations, is a shortcoming of our study, our systematic analysis and cross-validation of the bioanalytical methods allowed us to correct for this phenomenon and to perform an integral analysis of all PK and PD data on data of a rare disease. Nonetheless, our findings stress the necessity of cross-validation of laboratory methods (e.g. the quantification of eculizumab in serum when comparing results).

To evaluate our proposed dosing strategies of eculizumab, prospective validation of the non-inferiority of the proposed alternative dosing regimens is necessary before routinely implementing it in the clinic. Our developed PK–PD model may be implemented in existing model-informed precision dosing software for purposes of Bayesian dose individualization. This may facilitate implementation of eculizumab TDM in the clinic.

In conclusion, with our developed combined PK–PD model we showed that a weight-based loading dose of eculizumab, followed by PK-guided dosing, results in a more patient-friendly dosing regimen with the potential to improve treatment at reduced costs.

## Supplementary Material

gfac056_Supplemental_File

## APPENDIX

The CUREiHUS study group:

E. van Kempen and W. Altena, Dutch Kidney Patient Organisation, Bussum; E. Adang, Department of IQ Healthcare, Radboud University Medical Centre, Nijmegen; D.J.A.R. Moes, Department of Pharmacy, Leiden University Medical Centre, Leiden; A.D. van Zuijlen, Department of Nephrology, University Medical Centre Utrecht, Utrecht; S.P. Berger, Department of Nephrology, University Medical Centre Groningen, Groningen; F.J. Bemelman, Department of Nephrology, Amsterdam University Medical Centre, Amsterdam; J.W. van der Heijden, Department of Nephrology, Amsterdam University Medical Centre, Amsterdam; J. van de Wetering, Department of Nephrology, Erasmus Medical Centre, Rotterdam; A.P.J. de Vries, Department of Nephrology, Leiden University Medical Centre, Leiden; P. van Paasen, Department of Nephrology/Immunology, Maastricht University Medical Centre, Maastricht; J.F.M. Wetzels, Department of Nephrology, Radboud University Medical Centre, Nijmegen; J.A.E. van Wijk, Department of Paediatric Nephrology, Amsterdam University Medical Centre, Amsterdam; A.H.M. Bouts, Department of Paediatric Nephrology, Amsterdam University Medical Centre, Amsterdam; E.M. Dorresteijn, Department of Paediatric Nephrology, Sophia Children's Hospital, Erasmus Medical Centre, Rotterdam; V. Gracchi, Department of Paediatric Nephrology, University Medical Centre Groningen, Groningen; F.A.P.T. Horuz Engels, Department of Paediatric Nephrology, Maastricht University Medical Centre, Maastricht; M.G. Keijzer-Veen, Department of Paediatric Nephrology, Wilhelmina Children's Hospital, University Medical Centre Utrecht, Utrecht; R.W.G. van Rooij, Department of Paediatric Nephrology, Leiden University Medical Centre, Leiden; and N.C.A.J. van de Kar, Department of Paediatric Nephrology, Amalia Children's Hospital, Radboud University Medical Centre, Nijmegen.
