# Supplementary material for: Proposal for individualized dosing of eculizumab in atypical haemolytic uraemic syndrome: patient friendly and cost-effective
Source: Nephrol Dial Transplant. 2022 Mar 3;38(2):362–71. doi: 10.1093/ndt/gfac056 (PMC9923710; doi:10.1093/ndt/gfac056)
Supplement: gfac056_Supplemental_File [file gfac056_supplemental_file.docx]

**Proposal for individualized dosing of eculizumab in atypical hemolytic uremic syndrome: Patient friendly and cost-effective**

**SUPPLEMENTAL MATERIAL**

TABLE of CONTENTS
1. Bioanalysis of eculizumab concentrations and classical pathway (CP) activity 2
2. Development of a pharmacokinetic model 2
3. Development of a sequential pharmacokinetic-pharmacodynamic model 4
4. Figures 5
5. Nonmem code pharmacokinetic model 9
6. Nonmem code sequential pharmacokinetic-pharmacodynamic model 14
7. Literature 15

1. **Bioanalysis of eculizumab concentrations and classical pathway (CP) activity**
   1. *Method comparison*

Method comparison for both the eculizumab assays and the CP activity assays was done pair-wise for available paired data. Passing and Bablok regression analysis was used to determine slope and intercept. Bland Altman plots were used to measure agreement between the methods. See Figure S1 and S2.

1. **Development of a pharmacokinetic model – detailed methods and results**
   1. Methods

Population pharmacokinetic modelling was performed with NONMEM V7.4 (ICON Development Solutions, Ireland), Pearl-speaks-NONMEM (PsN) 4.8.0 and visualized using Pirana 2.9.7 (Pirana Software & Consulting BV) and R 3.6.10 ^1,2^. The first order conditional estimation method with interaction was used for the analysis. R (version 3.6.10) was used for the graphical analysis. Free eculizumab concentrations below limit of quantitation (BLQ) were discarded form the dataset, as they comprised only 3.7% of the total data ^3^. Inter-individual variability (IIV) and inter-occasion variability (IOV) were tested on all pharmacokinetic parameters. Residual unexplained variability (RUV) was tested with proportional and combined proportional and additional error models. Based on physiological plausibility and extensive previous evidence, allometric scaling was *a priori* used to account for the impact of weight on pharmacokinetics. Standard fixed exponent values of 0.75 for the flow dependent physiological process parameters and 1 for volume related parameters were used. Both total body weight and fat-free mass were tested for allometric scaling^4^. Model selection was initially based on comparing the objective function value (OFV), computed as −2 log likelihood, between nested models. A difference in OFV of ≥3.84, corresponding with a p value <0.05 for one degree of freedom, was considered statistically significant. The relative standard errors (RSE) on our parameters were based on the covariance step in NONMEM. In addition, standard goodness-of-fit (GOF)-plots, including observed versus population and individual predicted values and conditional weighted residuals versus time after dose or population predicted values were used for model evaluation. For the internal validation of the final model, a prediction-corrected visual predictive check (pcVPC) based on 1000 Monte Carlo simulations was performed^5^.

- 1. Results

A one-compartment model with parallel first order and Michaelis Menten elimination and a combined proportional and additive residual error model best fitted the data. Bodyweight and fat free mass with fixed allometric exponents were both a priori tested in the model on clearance (CL), volume of distribution (V) and maximum rate (V_max_). There was no significant difference in OFV between allometric scaling on total body weight versus fat free mass, so body weight was used in the final model and all pharmacokinetic parameters were scaled to a standard body weight of 70 kg. IIV on clearance and volume of distribution further improved the model, as well as IOV on clearance. Parameter estimates of the final model are summarized in Table 3. CL, V_d_, V_max_ and plasma concentration for 50% of maximum rate (K_m_) were estimated to be 0.163 L/day (RSE% 7.5), 6.42 L (5.9), 29.6 mg/day (7.0) and 37.9 mg/L (18.7). The IIV (%CV) on CL and V were estimated to be 43.4% and 37.1% respectively and IOV (%CV) on CL was estimated to be 34.4%. The proportional error component of RUV was 0.0247 for our in-house eculizumab assay and 0.248 for the Sanquin assay. The additional error was estimated to be 4.33 mg/L.

The standard goodness of fit plots of the final model did not show structural bias or major deviations (Figure S3). In addition, the pcVPC showed that most of the observations were within the 90% prediction interval and no deviating trends were observed, suggesting a good predictive performance of the final model (Figure 2).

1. **Development of a sequential pharmacokinetic-pharmacodynamic model – detailed methods and results**
   1. Methods

After the development of the pharmacokinetic model, a sequential pharmacokinetic-pharmacodynamic model was developed to describe the relationship between free eculizumab concentrations and the degree of complement blockade displayed as CP activity. We used an inhibitory E_max_ model to construct this relationship. Standard goodness-of-fit (GOF)-plots were used for model evaluation. To account for CP-activity data below the limit of quantitation (<10%) , we included these data using the M3 method as proposed by Beal et al^3^.

For the internal validation of the final model, a prediction-corrected visual predictive check (pcVPC) based on 1000 Monte Carlo simulations was performed^5^.

- 1. Results

Parameter estimates of the final model are summarized supplementary file Table 4. Baseline, I_max_, IC_50_ and γ were estimated to be 101% (RSE% 6.2), 0.959 (0.2), 22.0 mg/L (8.6) and 5.42 (4.6). The standard goodness of fit plots of the final model did not show structural bias or major deviations (Figure S4). In addition, the pcVPC showed that most of the observations were within the 90% prediction interval and no deviating trends were observed, suggesting a good internal predictive performance of the final model (Figure 3).

**Figures**


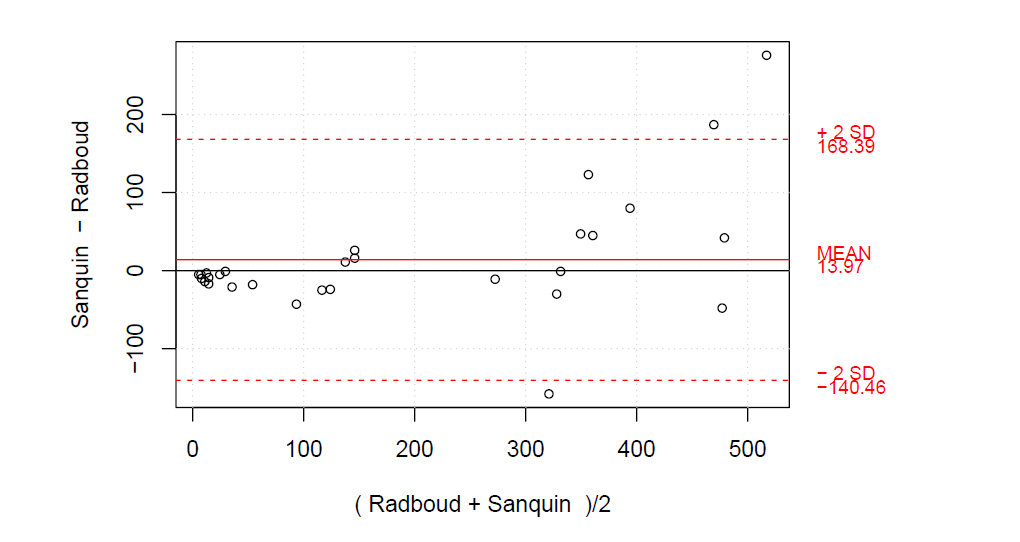


Figure S1 Bland-Altman plot for the Eculizumab assays. This plot shows the mean of the Radboud and Sanquin assay (x-axis) versus the difference between these assays (y-axis). SD = standard deviation.


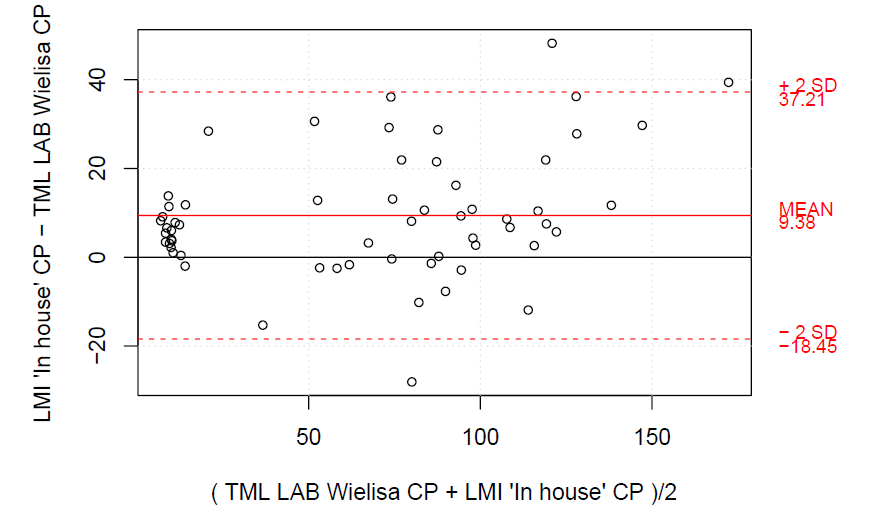


Figure S2 Bland-Altman plot for CP activity assays. This plot shows the mean of the TML lab Wielisa and LMI ‘in house’ assay (x-axis) versus the difference between these assays (y-axis). SD = standard deviation.


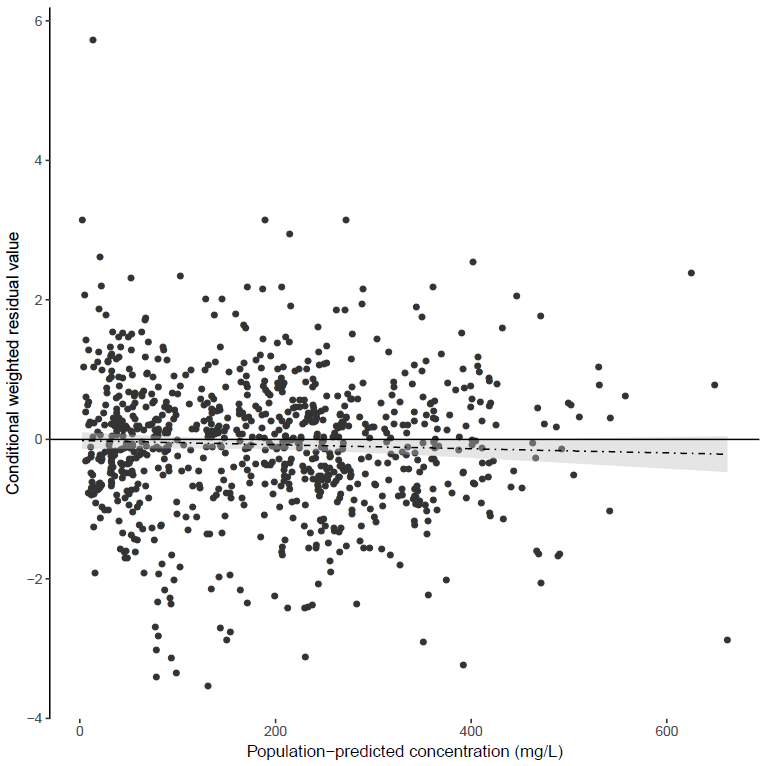

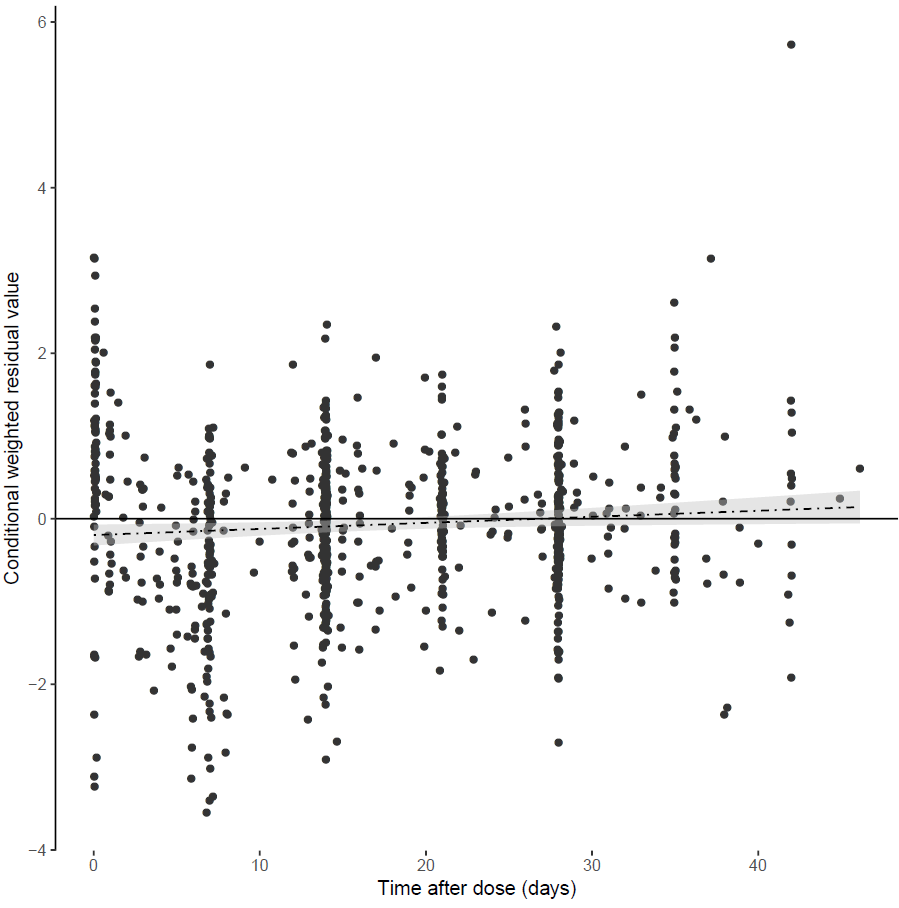


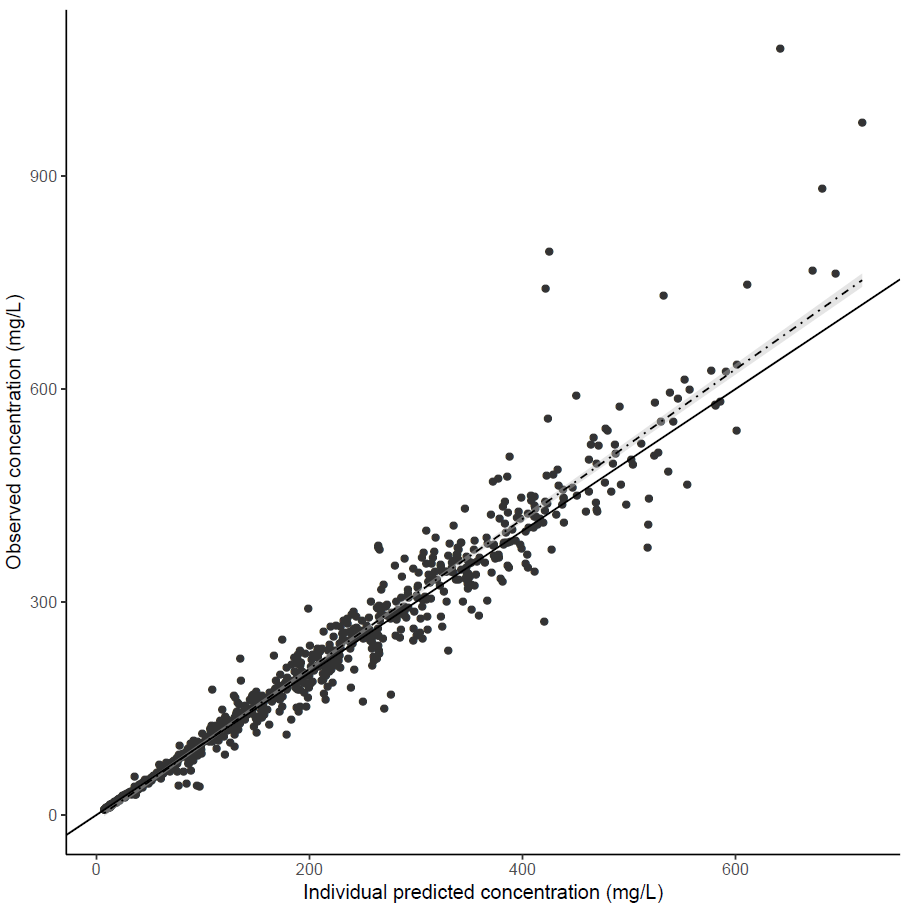

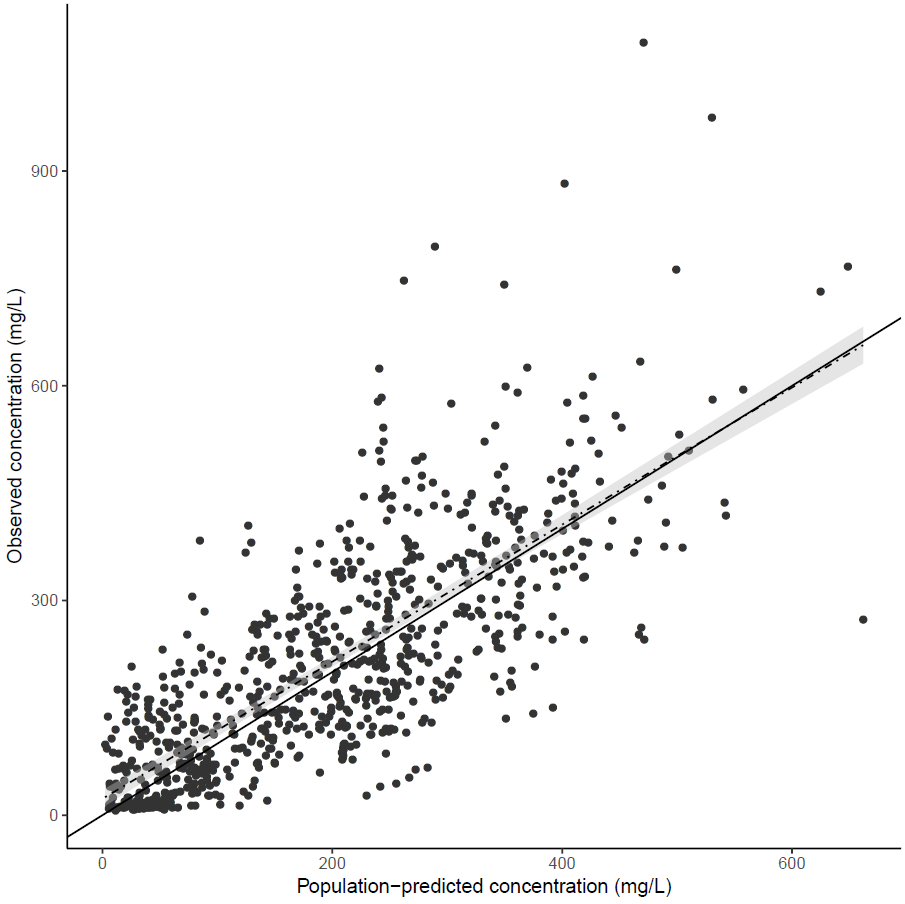


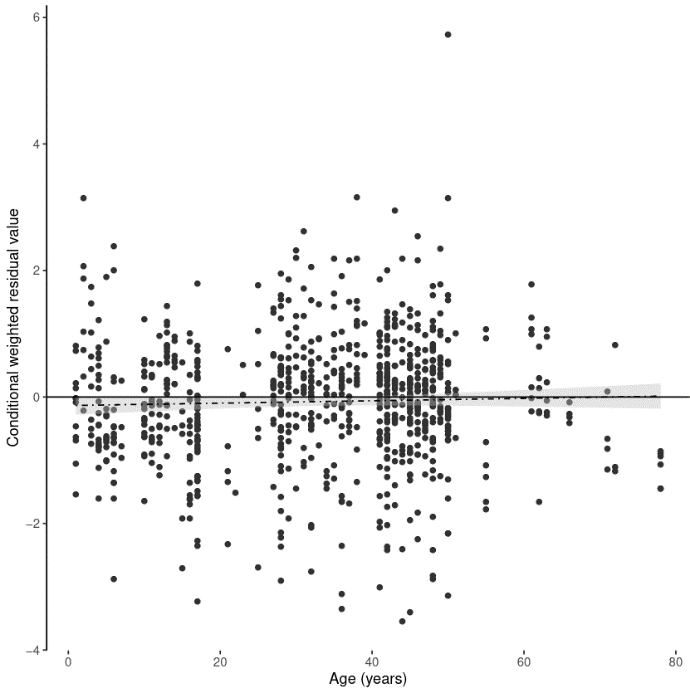

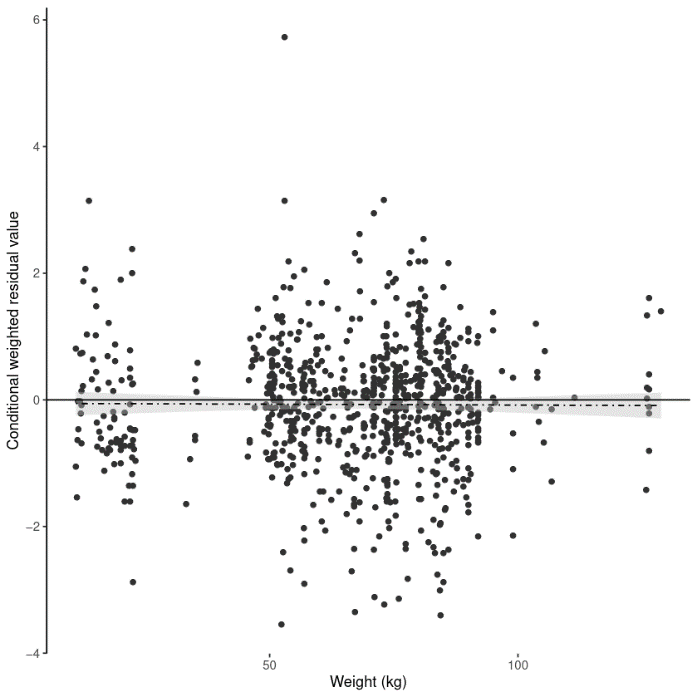


Figure S3. Standard goodness-of-fit plots for the final pharmacokinetic model of Eculizumab. The upper figures show the population predicted concentration (PRED) versus conditional weighted residuals (CWRES) and the time after dose (TAD) versus CWRES, respectively. The CWRES do not indicate model misspecification, as the data is homogenous distributed with the trendline approximating zero and most of the data lying within a -3 to +3 interval. The figures in the middle visualized the good correlation for the population predicted and individual predicted Eculizumab concentrations versus the observed concentrations. The bottom figures show the CWRES vs weight plot and CWRES vs age plot. The CWRES vs weight plot shows that we can predict the eculizumab pharmacokinetics over the complete weight range. The CWRES vs age plot shows that weight as a covariate in our model accounted for all age-dependent differences in pharmacokinetics of eculizumab. Both CWRES plots do not indicate model misspecification, as the data is homogenous distributed with the trendline approximating zero and most of the data lying within a -3 to +3 interval.


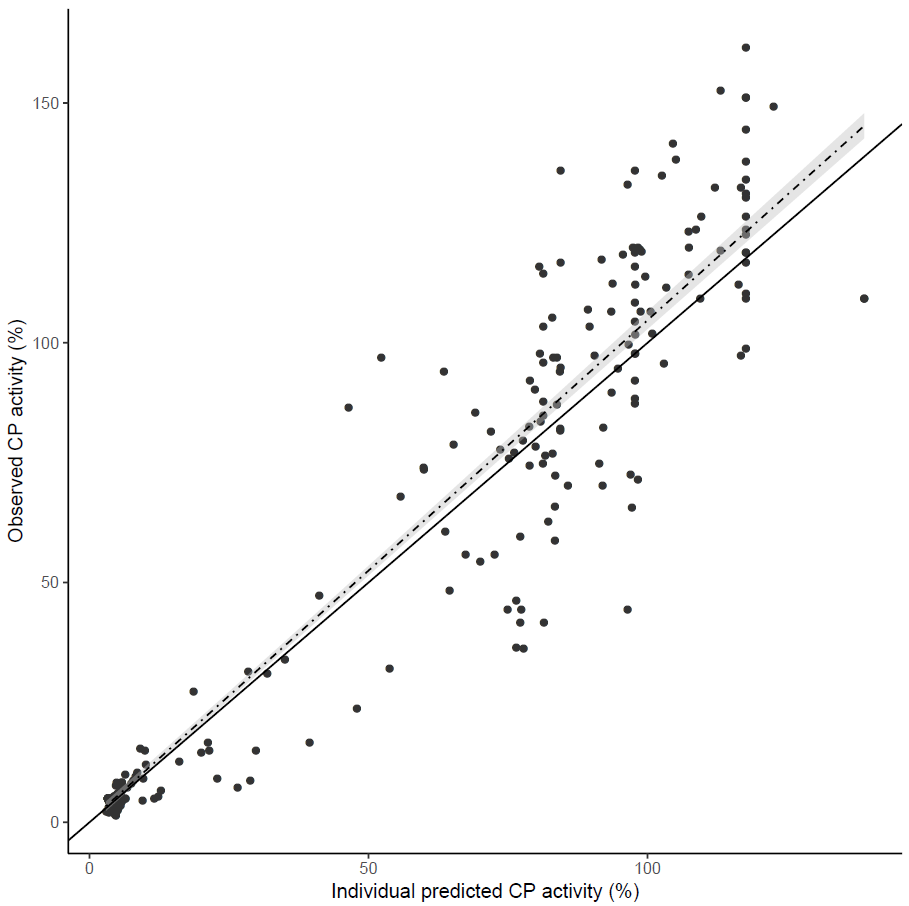

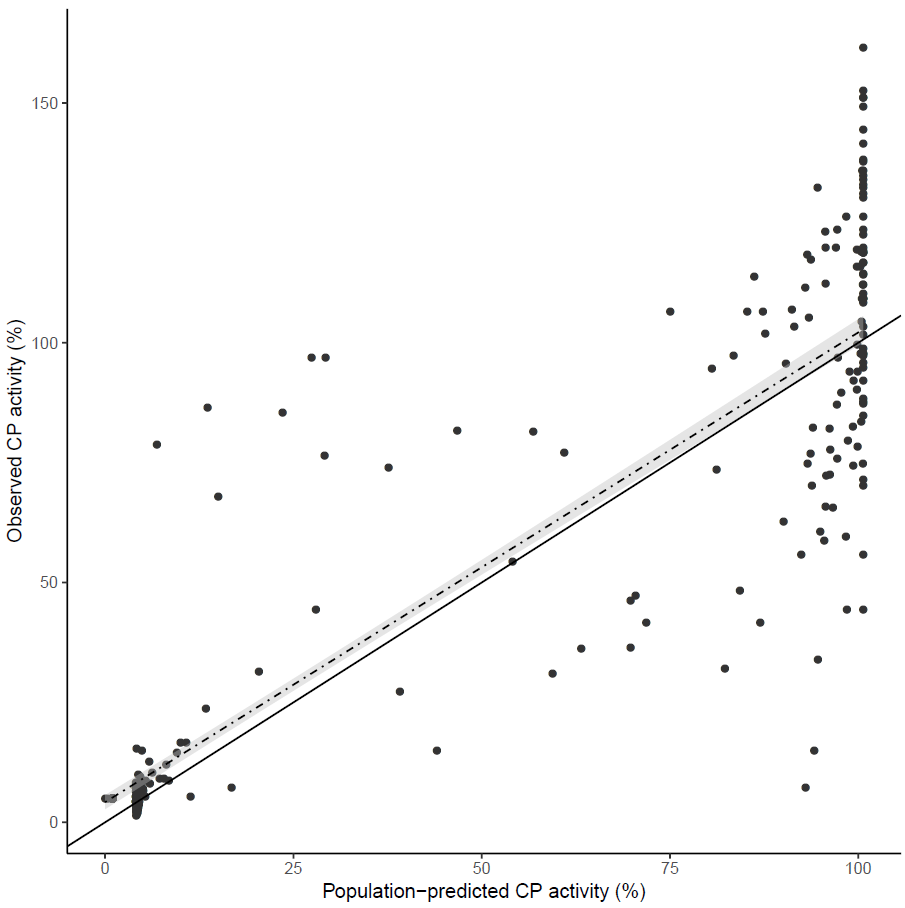


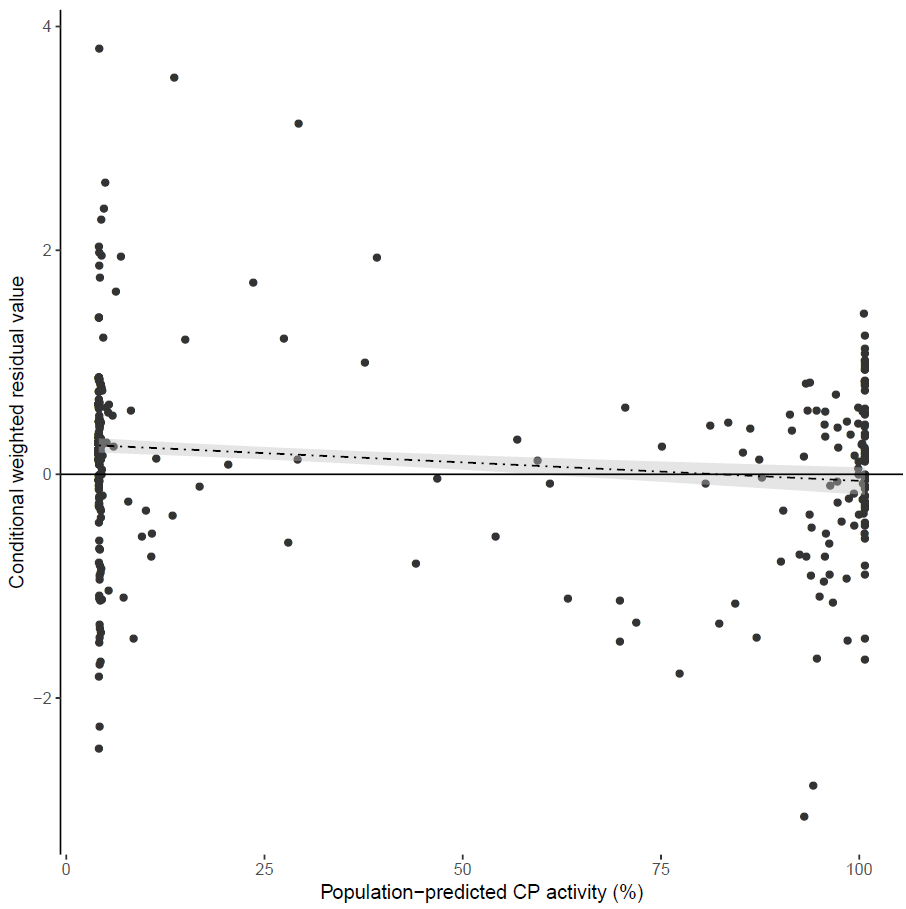


Figure S4. Standard goodness-of-fit plots for the final sequential pharmacokinetic-pharmacodynamic model. The upper panels visualized the good correlation for the population predicted and individual predicted CP activity versus the observed CP activity. The lower panel show the population predicted concentration (PRED) versus conditional weighted residuals (CWRES). The CWRES do not indicate model misspecification, as the data is homogenous distributed with the trendline approximating zero and most of the data lying within a -3 to +3 interval.

**Nonmem code pharmacokinetic model**

$SIZES LVR=91 LNP4=7000

$PROBLEM PK

$INPUT ID TIME AMT RATE DV DROP MDV EVID CMT WT HT SEX AGE II SS CH50 OCC METH1 METH2 TAFD

$DATA PKPD23.1.2020.csv IGNORE=@

$SUBROUTINES ADVAN13 TOL=6

$ABBREVIATED DERIV2=NOCOMMON

$MODEL COMP=(CENTRAL)

$PK

"FIRST

" COMMON /PRCOMG/ IDUM1,IDUM2,IMAX,IDUM4,IDUM5

" INTEGER IDUM1,IDUM2,IMAX,IDUM4,IDUM5

" IMAX=1000000

; --- TIME AFTER DOSE CALCULATION

IF (NEWIND.LE.1) THEN

DOSE=0

TDOS=0

ENDIF

;Remember dose and time of dose

IF (AMT.GT.0) THEN

DOSE=AMT

TDOS=TIME

ENDIF

;Time after dose for every record

TAD=TIME-TDOS

; IOV

IF(OCC.EQ.1) IOV = ETA(1)

IF(OCC.EQ.2) IOV = ETA(2)

IF(OCC.EQ.3) IOV = ETA(3)

IF(OCC.EQ.4) IOV = ETA(4)

IF(OCC.EQ.5) IOV = ETA(5)

IF(OCC.EQ.6) IOV = ETA(6)

IF(OCC.EQ.7) IOV = ETA(7)

IF(OCC.EQ.8) IOV = ETA(8)

IF(OCC.EQ.9) IOV = ETA(9)

IF(OCC.EQ.10) IOV = ETA(10)

IF(OCC.EQ.11) IOV = ETA(11)

IF(OCC.EQ.12) IOV = ETA(12)

IF(OCC.EQ.13) IOV = ETA(13)

IF(OCC.EQ.14) IOV = ETA(14)

IF(OCC.EQ.15) IOV = ETA(15)

IF(OCC.EQ.16) IOV = ETA(16)

IF(OCC.EQ.17) IOV = ETA(17)

IF(OCC.EQ.18) IOV = ETA(18)

IF(OCC.EQ.19) IOV = ETA(19)

IF(OCC.EQ.20) IOV = ETA(20)

IF(OCC.EQ.21) IOV = ETA(21)

IF(OCC.EQ.22) IOV = ETA(22)

IF(OCC.EQ.23) IOV = ETA(23)

IF(OCC.EQ.24) IOV = ETA(24)

IF(OCC.EQ.25) IOV = ETA(25)

IF(OCC.EQ.26) IOV = ETA(26)

IF(OCC.EQ.27) IOV = ETA(27)

IF(OCC.EQ.28) IOV = ETA(28)

IF(OCC.EQ.29) IOV = ETA(29)

IF(OCC.EQ.30) IOV = ETA(30)

IF(OCC.EQ.31) IOV = ETA(31)

IF(OCC.EQ.32) IOV = ETA(32)

IF(OCC.EQ.33) IOV = ETA(33)

IF(OCC.EQ.34) IOV = ETA(34)

IF(OCC.EQ.35) IOV = ETA(35)

IF(OCC.EQ.36) IOV = ETA(36)

IF(OCC.EQ.37) IOV = ETA(37)

IF(OCC.EQ.38) IOV = ETA(38)

IF(OCC.EQ.39) IOV = ETA(39)

IF(OCC.EQ.40) IOV = ETA(40)

IF(OCC.EQ.41) IOV = ETA(41)

IF(OCC.EQ.42) IOV = ETA(42)

IF(OCC.EQ.43) IOV = ETA(43)

IF(OCC.EQ.44) IOV = ETA(44)

IF(OCC.EQ.45) IOV = ETA(45)

IF(OCC.EQ.46) IOV = ETA(46)

IF(OCC.EQ.47) IOV = ETA(47)

IF(OCC.EQ.48) IOV = ETA(48)

IF(OCC.EQ.49) IOV = ETA(49)

IF(OCC.EQ.50) IOV = ETA(50)

IF(OCC.EQ.51) IOV = ETA(51)

IF(OCC.EQ.52) IOV = ETA(52)

IF(OCC.EQ.53) IOV = ETA(53)

IF(OCC.EQ.54) IOV = ETA(54)

IF(OCC.EQ.55) IOV = ETA(55)

IF(OCC.EQ.56) IOV = ETA(56)

IF(OCC.EQ.57) IOV = ETA(57)

IF(OCC.EQ.58) IOV = ETA(58)

IF(OCC.EQ.59) IOV = ETA(59)

IF(OCC.EQ.60) IOV = ETA(60)

IF(OCC.EQ.61) IOV = ETA(61)

IF(OCC.EQ.62) IOV = ETA(62)

IF(OCC.EQ.63) IOV = ETA(63)

IF(OCC.EQ.64) IOV = ETA(64)

IF(OCC.EQ.65) IOV = ETA(65)

IF(OCC.EQ.66) IOV = ETA(66)

IF(OCC.EQ.67) IOV = ETA(67)

IF(OCC.EQ.68) IOV = ETA(68)

IF(OCC.EQ.69) IOV = ETA(69)

IF(OCC.EQ.70) IOV = ETA(70)

IF(OCC.EQ.71) IOV = ETA(71)

IF(OCC.EQ.72) IOV = ETA(72)

IF(OCC.EQ.73) IOV = ETA(73)

IF(OCC.EQ.74) IOV = ETA(74)

IF(OCC.EQ.75) IOV = ETA(75)

IF(OCC.EQ.76) IOV = ETA(76)

IF(OCC.EQ.77) IOV = ETA(77)

IF(OCC.EQ.78) IOV = ETA(78)

IF(OCC.EQ.79) IOV = ETA(79)

IF(OCC.EQ.80) IOV = ETA(80)

IF(OCC.EQ.81) IOV = ETA(81)

IF(OCC.EQ.82) IOV = ETA(82)

IF(OCC.EQ.83) IOV = ETA(83)

IF(OCC.EQ.84) IOV = ETA(84)

IF(OCC.EQ.85) IOV = ETA(85)

IF(OCC.EQ.86) IOV = ETA(86)

CL = THETA(1) * ((WT/70)**0.75) * EXP(ETA(87)) * EXP(IOV)

V1 = THETA(2) * (WT/70) * EXP(ETA(88))

VM = THETA(3) * (WT/70)**0.75

KM = THETA(4)

ICL=CL

IV1=V1

V=V1

S1=V

KEL=CL/V1

$ERROR

IPRED=F

IF (METH1.LT.2) Y=IPRED+ERR(1)+(IPRED*ERR(2))

IF (METH1.EQ.2) Y=IPRED+ERR(1)+(IPRED*ERR(3))

$THETA

(0.163) ; 1 CL

(6.42) ; 2 V1

(29.6) ; 3 VM

(37.9) ; 4 KM

$DES

C1=A(1)/S1

DADT(1)=- KEL*A(1) - ((VM*C1)/(KM+C1))

$OMEGA BLOCK(1) 0.1 ; 1 IOV

$OMEGA BLOCK(1) SAME ; 2 -86 IOV

$OMEGA BLOCK(1) SAME ; 2 -86 IOV

$OMEGA BLOCK(1) SAME ; 2 -86 IOV

$OMEGA BLOCK(1) SAME ; 2 -86 IOV

$OMEGA BLOCK(1) SAME ; 2 -86 IOV

$OMEGA BLOCK(1) SAME ; 2 -86 IOV

$OMEGA BLOCK(1) SAME ; 2 -86 IOV

$OMEGA BLOCK(1) SAME ; 2 -86 IOV

$OMEGA BLOCK(1) SAME ; 2 -86 IOV

$OMEGA BLOCK(1) SAME ; 2 -86 IOV

$OMEGA BLOCK(1) SAME ; 2 -86 IOV

$OMEGA BLOCK(1) SAME ; 2 -86 IOV

$OMEGA BLOCK(1) SAME ; 2 -86 IOV

$OMEGA BLOCK(1) SAME ; 2 -86 IOV

$OMEGA BLOCK(1) SAME ; 2 -86 IOV

$OMEGA BLOCK(1) SAME ; 2 -86 IOV

$OMEGA BLOCK(1) SAME ; 2 -86 IOV

$OMEGA BLOCK(1) SAME ; 2 -86 IOV

$OMEGA BLOCK(1) SAME ; 2 -86 IOV

$OMEGA BLOCK(1) SAME ; 2 -86 IOV

$OMEGA BLOCK(1) SAME ; 2 -86 IOV

$OMEGA BLOCK(1) SAME ; 2 -86 IOV

$OMEGA BLOCK(1) SAME ; 2 -86 IOV

$OMEGA BLOCK(1) SAME ; 2 -86 IOV

$OMEGA BLOCK(1) SAME ; 2 -86 IOV

$OMEGA BLOCK(1) SAME ; 2 -86 IOV

$OMEGA BLOCK(1) SAME ; 2 -86 IOV

$OMEGA BLOCK(1) SAME ; 2 -86 IOV

$OMEGA BLOCK(1) SAME ; 2 -86 IOV

$OMEGA BLOCK(1) SAME ; 2 -86 IOV

$OMEGA BLOCK(1) SAME ; 2 -86 IOV

$OMEGA BLOCK(1) SAME ; 2 -86 IOV

$OMEGA BLOCK(1) SAME ; 2 -86 IOV

$OMEGA BLOCK(1) SAME ; 2 -86 IOV

$OMEGA BLOCK(1) SAME ; 2 -86 IOV

$OMEGA BLOCK(1) SAME ; 2 -86 IOV

$OMEGA BLOCK(1) SAME ; 2 -86 IOV

$OMEGA BLOCK(1) SAME ; 2 -86 IOV

$OMEGA BLOCK(1) SAME ; 2 -86 IOV

$OMEGA BLOCK(1) SAME ; 2 -86 IOV

$OMEGA BLOCK(1) SAME ; 2 -86 IOV

$OMEGA BLOCK(1) SAME ; 2 -86 IOV

$OMEGA BLOCK(1) SAME ; 2 -86 IOV

$OMEGA BLOCK(1) SAME ; 2 -86 IOV

$OMEGA BLOCK(1) SAME ; 2 -86 IOV

$OMEGA BLOCK(1) SAME ; 2 -86 IOV

$OMEGA BLOCK(1) SAME ; 2 -86 IOV

$OMEGA BLOCK(1) SAME ; 2 -86 IOV

$OMEGA BLOCK(1) SAME ; 2 -86 IOV

$OMEGA BLOCK(1) SAME ; 2 -86 IOV

$OMEGA BLOCK(1) SAME ; 2 -86 IOV

$OMEGA BLOCK(1) SAME ; 2 -86 IOV

$OMEGA BLOCK(1) SAME ; 2 -86 IOV

$OMEGA BLOCK(1) SAME ; 2 -86 IOV

$OMEGA BLOCK(1) SAME ; 2 -86 IOV

$OMEGA BLOCK(1) SAME ; 2 -86 IOV

$OMEGA BLOCK(1) SAME ; 2 -86 IOV

$OMEGA BLOCK(1) SAME ; 2 -86 IOV

$OMEGA BLOCK(1) SAME ; 2 -86 IOV

$OMEGA BLOCK(1) SAME ; 2 -86 IOV

$OMEGA BLOCK(1) SAME ; 2 -86 IOV

$OMEGA BLOCK(1) SAME ; 2 -86 IOV

$OMEGA BLOCK(1) SAME ; 2 -86 IOV

$OMEGA BLOCK(1) SAME ; 2 -86 IOV

$OMEGA BLOCK(1) SAME ; 2 -86 IOV

$OMEGA BLOCK(1) SAME ; 2 -86 IOV

$OMEGA BLOCK(1) SAME ; 2 -86 IOV

$OMEGA BLOCK(1) SAME ; 2 -86 IOV

$OMEGA BLOCK(1) SAME ; 2 -86 IOV

$OMEGA BLOCK(1) SAME ; 2 -86 IOV

$OMEGA BLOCK(1) SAME ; 2 -86 IOV

$OMEGA BLOCK(1) SAME ; 2 -86 IOV

$OMEGA BLOCK(1) SAME ; 2 -86 IOV

$OMEGA BLOCK(1) SAME ; 2 -86 IOV

$OMEGA BLOCK(1) SAME ; 2 -86 IOV

$OMEGA BLOCK(1) SAME ; 2 -86 IOV

$OMEGA BLOCK(1) SAME ; 2 -86 IOV

$OMEGA BLOCK(1) SAME ; 2 -86 IOV

$OMEGA BLOCK(1) SAME ; 2 -86 IOV

$OMEGA BLOCK(1) SAME ; 2 -86 IOV

$OMEGA BLOCK(1) SAME ; 2 -86 IOV

$OMEGA BLOCK(1) SAME ; 2 -86 IOV

$OMEGA BLOCK(1) SAME ; 2 -86 IOV

$OMEGA BLOCK(1) SAME ; 2 -86 IOV

$OMEGA BLOCK(1) SAME ; 2 -86 IOV

$OMEGA

0.188 ; IIV CL

0.138 ; IIV V1

$SIGMA

4.33 ; ADD

0.0247; PROP

0.248 ; PROP2

$EST METHOD=1 INTERACTION NOABORT MAXEVAL=5000 NSIG=2 SIGL=6 PRINT=5

$COVARIANCE PRINT=E MATRIX=R

$TABLE ID TIME TAD AMT RATE DV MDV EVID CMT WT FFM HT SEX AGE II SS CH50 OCC ICL IV1 IPRED PRED C1 TAFD METH1 METH2 CWRES CWRESI NPDE NOAPPEND NOPRINT ONEHEADER FILE=sdtabrun1a.tab

**Nonmem code sequential pharmacokinetic-pharmacodynamic model**

$SIZES LVR=85

$PROBLEM PK

$INPUT ID DV IPREDC METH

$DATA PD23.1.2020.csv IGNORE=@

$PRED

CONC=IPREDC

BLQ=0

IF (DV.EQ.5) BLQ=1 ; IF DV IS 5, DATA ARE BELOW LQ

IMAX=THETA(1)*EXP(ETA(1))

IC50=THETA(2)*EXP(ETA(2))

BASE=THETA(3)*EXP(ETA(3))

GAM=THETA(4)*EXP(ETA(4))

E=BASE*(1-((IMAX*CONC**GAM)/(IC50**GAM+CONC**GAM)))

IPRED=E

; --- CODE FOR M3 METHOD BY BEAL ET AL

IF (BLQ.EQ.0) THEN

F_FLAG=0

Y=IPRED+IPRED*ERR(1)

ELSE

F_FLAG=1

DUM=(10-IPRED)/(SQRT(SIGMA(1,1))*IPRED)

CUMD=PHI(DUM)

Y=CUMD

ENDIF

$THETA

(0.96); Imax

(22.0) ; IC50

(100.7) ; BASELINE CH50

(5.42); GAMMA (HILL)

$OMEGA

0 FIX ; IMAX

0.149 ; IC50

0.0529; BASE

0 FIX ; GAM

$SIGMA

0.089 ; PROP ERR

$EST METHOD=1 INTERACTION LAPLACIAN MAXEVAL=2000 NOABORT

$COV MATRIX=S PRINT=E

$TABLE ID DV CONC IPRED PRED CPREDI CWRES CWRESI CWRESI NPDE NOPRINT ONEHEADER FILE=sdtabPKPD.tab

**Literature**

1. Beal SL, Sheiner LB, Boeckmann A, Bauer RJ. NONMEM users guides. *NONMEM Project Group, University of California, San Francisco*. 1992.

2. Keizer R, Karlsson M, Hooker A. Modeling and simulation workbench for NONMEM: tutorial on Pirana, PsN, and Xpose. CPT Pharmacomet Syst Pharmacol 2: e50; 2013.

3. Beal SL. Ways to fit a PK model with some data below the quantification limit. *J Pharmacokinet Pharmacodyn*. 2001;28(5):481-504.

4. Anderson BJ, Holford NH. Mechanism-based concepts of size and maturity in pharmacokinetics. *Annu Rev Pharmacol Toxicol*. 2008;48:303-332.

5. Bergstrand M, Hooker AC, Wallin JE, Karlsson MO. Prediction-corrected visual predictive checks for diagnosing nonlinear mixed-effects models. *Aaps j*. 2011;13(2):143-151.
